# 基于超高效液相色谱-四极杆飞行时间质谱的非靶向代谢组学用于不同来源单花蜜的差异分析

**DOI:** 10.3724/SP.J.1123.2020.06029

**Published:** 2021-03-08

**Authors:** Shi SHEN, Yi YANG, Jingbo WANG, Xi CHEN, Tingting LIU, Qin ZHUO

**Affiliations:** 1.中国疾病预防控制中心营养与健康所, 国家卫生健康委员会微量元素重点实验室, 北京 100050; 1. National Institute for Nutrition and Health, Chinese Center for Disease Control and Prevention, Key Laboratory of Trace Element Nutrition, National Health Commission of the People’s Republic of China, Beijing 100050, China; 2.北京市疾病预防控制中心, 北京 100013; 2. Beijing Center for Disease Control and Prevention, Beijing 100013, China

**Keywords:** 超高效液相色谱, 四极杆飞行时间质谱, 植物代谢组学, 单花蜜, 化学计量学, 溯源识别, ultra-high performance liquid chromatography (UPLC), quadrupole time-of-flight mass spectrometry (Q-TOF-MS), plant metabolomics, unifloral honey, chemometrics, traceability recognition

## Abstract

不同的蜜源植物具有结构多样的次生代谢产物。该研究以8种不同蜜源单花蜜(洋槐蜜、枣花蜜、荆条蜜、椴树蜜、荞麦蜜、麦卢卡蜜、枸杞蜜、益母草蜜)为研究对象,建立了基于超高效液相色谱-四极杆飞行时间质谱技术(UPLC-Q-TOF-MS^E^)的非靶向代谢组学方法,考察了不同蜜源中次生代谢产物的差异。该研究采用固相萃取前处理方法和UPLC-Q-TOF-MS^E^方法,获得不同蜜源单花蜜的植物代谢组信息,并构建了多变量统计分析模型,对不同来源的单花蜜进行模式识别和差异分析,发现洋槐蜜、枣花蜜、荆条蜜、椴树蜜、荞麦蜜、麦卢卡蜜相互间存在不同程度的显著差异。结合模型的变量重要性投影、方差分析与最大差异倍数值,根据精确前体离子和碎片离子质量信息检索Chemspider、HMDB数据库,该研究筛选并鉴定出32个代谢差异化合物,其中黄酮类化合物18个、酚酸类化合物7个、苯苷与萜苷类化合物6个、甾体类化合物1个;研究发现麦卢卡蜜和荞麦蜜以黄酮类化合物为主要差异代谢物,荆条蜜中酚酸类化合物为特征性表达,苯苷与萜苷类化合物主要为椴树蜜的特征代谢物。该研究从植物代谢组学角度初步揭示了不同单花蜜的代谢产物差异性以及特征化合物,为基于化学分析技术的蜂蜜溯源识别与质量评价提供了有效的研究策略。

## Abstract (en)

Different nectar plants contain various secondary metabolites. Herein, the differences in the contents of endogenous metabolites in honeys from eight botanical origins (i. e., acacia, jujube, vitex, linden, buckwheat, manuka, wolfberry, and motherwort honeys) were investigated by a non-targeted metabolomics-based method. This method involved solid-phase extraction pretreatment and ultra-high performance liquid chromatography-quadrupole time-of-flight mass spectrometry (UPLC-Q-TOF-MS^E^). An oasis HLB cartridge was used for the removal of many saccharides. Chromatographic experiments were performed on an HSS T3 column (100 mm×2.1 mm, 1.8 μm) using a mobile phase that consisted of 0.1% (v/v) formic acid in acetonitrile and water. Mass spectrometry was conducted in the positive and negative modes by electrospray ionization (ESI). Metabolic information about the honeys from different botanical origins was acquired using a multivariate statistical analysis model. Principal component analysis (PCA) and partial least square discriminant analysis (PLS-DA) were conducted for pattern recognition and difference analysis. PCA was performed for 10557 and 2706 data variables in the positive and negative ion modes, respectively. The distribution of honeys from different botanical origins was investigated in 88 honey samples. The three principal components exhibited 48.05% and 57.88% of the total variance in positive and negative ion modes, respectively. The samples studied were divided into six different groups on the basis of their botanical origins and metabolic compounds: linden, vitex, buckwheat, manuka, jujube, and acacia honeys. A permutation test (*n*=200) was conducted to verify the fit of the model. The differential metabolites were screened on the basis of variable importance in project (VIP; >1), analysis of variance (ANOVA; *p*<0.05), and maximum fold change (>1.5) by using the PLS-DA model. The compounds were identified based on the data retrieved from the Chemspider and HMDB databases according to the quality information of precursor ions and fragment ions. Thirty-two differential metabolites were screened and primarily identified according to the characteristic fragmentation rules of specific structure types and data retrieval, including 18 flavonoids, 7 phenolic acids, 6 phenyl and terpenoid glycosides, and 1 steroid. Various flavonoids in buckwheat and manuka honeys, such as quercetin, sakuranetin, 7-hydroxy-2-(4-hydroxy-3,5-dimethoxyphenyl)-4*H*-chromen-4-one, 5,7-dihydroxy-2-(3-methoxyphenyl)-4*H*-chromen-4-one, luteolin-7-methyl ether, and pollenitin, were found. In buckwheat honey, the contents of 3-methoxy-2-(4-methylbenzoyl)-4*H*-chromen-4-one, 2-hydroxy-3,4-diphenylpentanedioic acid, 3'-methoxydihydroformononetin, phenylpyruvic acid, 2-*O-p*-coumaroyltartronic acid, 2-(3-hydroxy-4,5-dimethoxyphenyl)-4*H*-chromen-4-one, 7-hydroxy-6-methoxy-3-(4-methoxyphenyl)-4*H*-chromen-4-one, 4-[(2*E*)-3-(4-hydroxyphenyl)prop-2-en-1-yl]-3-methoxyphenol, and 7-hydroxy-5-methoxyflavan were the highest; these compounds are the characteristic metabolites of buckwheat honey. In addition, manuka honey possessed the highest contents of gnaphaliin and galangin 3-methyl ether. Moreover, linden honey contained the characteristic phenyl glycosides of (*S*)-multifidol 2-[apiosyl-(1➝6)-glucoside], 2-phenylethyl-*β*-D-glucopyranoside, benzyl *O*-[arabinofuranosyl-(1➝6)-glucoside], crosatoside B, and terpenoid glycosides of isopentyl gentiobioside and 6-*O*-oleuropeoylsucrose. Vitex honey was found to be rich in quinic acid derivatives such as caffeoyl-3-*O*-feruloyl-quinic acid/1-feruloyl-5-caffeoylquinic acid, 3-*O*-caffeoyl-4-*O*-methyl-quinic acid/3-feruloylquinic acid, and 3-*O*-caffeoyl-1-*O*-methyl-quinic acid, in addition to the flavonoids of vitexin, namely, 6″-(3-hydroxy-3-methylglutarate) and apigenin-7-[galactosyl-(1➝4)-mannoside]. Moreover, ponasteroside A was a characteristic marker of jujube honey, and the contents of 6-*C*-fucosylluteolin and kaempferol 3-(2″-rhamnosylrutinoside) were the highest in acacia honey. In conclusion, the method based on non-targeted metabolomics involving UPLC-Q-TOF-MS^E^ for different unifloral honeys was found to be fast, effective, specific, and accurate. The differences in metabolite contents and the characteristic compounds in various unifloral honeys were preliminarily illustrated. This study provides an effective analytical strategy for honey traceability and quality analysis of unifloral honey.

蜂蜜为蜜蜂科昆虫中华蜜蜂*Apis cerana Fabricius*或意大利蜂*Apis mellifera Linnaeus*所酿的蜜,在2020版《中国药典》中有收载^[1]^。蜜蜂的采集物源于蜜源植物,其中主要蜜源植物是指在养蜂生产上能采到大量商品蜜的植物,如荆条、洋槐、椴树等。我国不仅地域辽阔,而且气候类型多样,因此蜜源植物丰富,主要蜜源植物约有30种,其中不同的蜜种在颜色深浅、味道清香或浓郁、黏度等方面具有不同特性。此外我国具有悠久的中药栽培历史,药用蜜源植物种类有数百种,有些种类可采到商品蜜,如枸杞蜜、益母草蜜、黄芪蜜等都是畅销蜜种^[2]^。由于不同的蜂蜜依据产量、口感、功效的差异价格悬殊,因此建立有效的蜂蜜蜜源识别方法对蜂蜜的质量评价非常必要。

不同的蜜源植物具有结构多样的次生代谢产物,主要分为挥发性小分子和萜类、黄酮类、酚酸类和生物碱类化合物,其通过蜜蜂酿造转化成蜂蜜中的特征代谢产物。蜂蜜中的特征代谢产物受植物源的生物合成途径、地理气候、储存方式等多种因素的影响,可作为判别蜜源、区别不同产地的依据^[3,4]^。研究发现黄酮类化合物橙皮素是柑橘蜂蜜的特征标志物^[5]^; Truchado等^[6]^在2008年首次在刺槐蜂蜜中发现了8个黄酮苷(山柰素的鼠李糖和己糖苷类化合物)。

溯源检测技术在蜂蜜品种鉴定中起到重要的技术支持作用,对比传统的感官鉴定和花粉分析法,目前新的分析技术如色谱^[7,8]^、质谱^[9,10,11,12,13]^、近红外光谱^[14]^、拉曼光谱^[15]^、核磁共振波谱^[16,17]^、电感耦合等离子体质谱^[18]^和分子生物学^[19]^等正逐渐被应用于蜜源鉴别。基于蜂蜜理化分析数据,结合多变量统计分析模型对蜂蜜化学成分进行差异分析从而判断蜜源正逐渐成为国内外蜂蜜品种鉴别的热门研究方法。近年来基于液相色谱-四极杆飞行时间质谱(LC-Q-TOF-MS)技术的代谢组学分析在食品物种及品种鉴定、产地鉴定、品质鉴别、掺假掺杂鉴定等方面应用广泛,体现了其他检测手段无法比拟的优势^[20]^。研究发现运用LC-Q-TOF-MS技术全面获取蜂蜜中所有代谢产物的化学信息并基于代谢组学进行差异分析具有分析快速且能鉴别未知标志物的优点。目前国内沈崇钰等^[12]^和严丽娟等^[13]^分别建立了基于液相色谱-高分辨质谱的代谢组学技术用于麦卢卡蜂蜜的甄别,国外Jandríc等^[11]^开展了基于LC-Q-TOF-MS技术对澳大利亚产的不同来源的蜂蜜差异分析研究。因此,面对我国丰富的单花蜜资源,基于LC-Q-TOF-MS技术建立代谢组学分析方法,可作为蜂蜜溯源识别的有效研究策略。

本研究收集了我国市场上常见的8种不同来源的蜂蜜(洋槐蜂蜜、枣花蜂蜜、荆条蜂蜜、椴树蜂蜜、枸杞蜂蜜、益母草蜂蜜、荞麦蜂蜜、麦卢卡蜂蜜)共88个样本。采用固相萃取-超高效液相色谱-四极杆飞行时间质谱(SPE-UPLC-Q-TOF-MS)技术,结合化学计量学统计方法,对不同蜜源蜂蜜进行差异识别,并检索相关数据库进行差异代谢物的结构推测,从植物代谢组学角度初步揭示了不同单花蜜的蜜源差异性以及特征化合物,为基于化学分析技术的蜂蜜溯源识别与质量评价提供了有效的研究策略。

## 1 实验部分

### 1.1 仪器、试剂与材料

Acquity H-Class超高效液相色谱仪串联SYNAPT G2-Si飞行时间质谱仪,配Masslynx质谱软件及Progenesis QI数据处理软件,均来自美国Waters科技有限公司;KQ5200E型数控超声波清洗仪(昆山市仪器超声有限公司); Milli-Q超纯水制备系统(美国Millipore公司); ME403E电子分析天平(瑞士Mettler Toledo公司,精确至0.001 g);旋转蒸发仪(德国IKA公司); Oasis HLB固相萃取柱(6 mL, 1 g)和微孔滤膜0.22 μm均由美国Waters公司提供。

甲醇(色谱纯级),乙腈、甲酸(质谱级)均购自Fisher Scientific公司; 槲皮素(纯度98.71%)购自Sigma Aldrich公司,樱花亭(纯度98.0%)购自深圳浩博世纪生物有限公司。

### 1.2 实验方法

1.2.1 样品采集与制备

所有蜂蜜样本2017-2019年采集于北京市场,共88个商品蜜样品,分别为12个洋槐蜜(北京、山东)、11个枣花蜜(北京、陕西)、9个荆条蜜(北京、山东)、20个椴树蜜(黑龙江、吉林、俄罗斯)、9个枸杞蜂蜜(北京、宁夏)、7个益母草蜂蜜(上海、北京、浙江、江西)、9个荞麦蜜(陕西、甘肃)和11个麦卢卡蜂蜜(新西兰)。所有的蜂蜜样品均在4 ℃的冰箱中贮藏待实验用。

称取蜂蜜样品10 g(精确至0.01 g),加入50 mL酸水溶液(1 mol/L盐酸调pH 2,下同),涡旋混匀后超声提取30 min, 10000 r/min离心5 min,取上清液待净化。Oasis HLB固相萃取小柱分别用5 mL甲醇和15 mL酸水溶液依次活化和预平衡后,将样品上清液转移至固相萃取小柱,待样品试液全部流出后,先用50 mL酸水溶液淋洗小柱除去糖及大极性分子,再用50 mL甲醇洗脱,收集洗脱液在40 ℃减压旋转蒸发浓缩至干,用1 mL甲醇复溶,经0.22 μm有机滤膜过滤,待上机测定^[10]^。

质量控制(quality control, QC)样品为8种单花蜜样品前处理后再等量混合得到的样品,用于监测分析系统的稳定性。

1.2.2 色谱条件及分析顺序

采用Acquity UPLC HSS T3色谱柱(100 mm×2.1 mm, 1.8 μm),以0.1%甲酸-水溶液为流动相A, 0.1%甲酸乙腈溶液为流动相B,按照体积比进行梯度洗脱:0~1.0 min, 97%A; 1.0~18.0 min, 97%A~10%A; 18.0~20.0 min, 10%A; 20.0~20.1 min, 10%A~0%A;流速0.3 mL/min,柱温35 ℃,进样量5 μL。

分析样品时先进3次空白溶剂样品,再连续进样10次QC样品,待仪器检测稳定后,开始实际样品检测。实际样品随机排列,每进样15次实际样品插入1次QC样品。

1.2.3 质谱条件

采用电喷雾正离子和负离子检测电离模式,雾化气为高纯度氮气,碰撞气为高纯度氦气,质量扫描范围:*m/z* 50~1200,采集模式为MS^E^灵敏度模式;正离子模式锥孔电压为40 V,毛细管电压为3.0 kV,离子源温度120 ℃,脱溶剂气温度450 ℃,脱溶剂气体积流量900 L/h,锥孔气体积流量50 L/h,碰撞能量(CE)10~45 eV, LockMass:亮氨酸脑啡肽(*m/z* 556.2771);负离子模式锥孔电压为40 V,毛细管电压为2.5 kV,离子源温度120 ℃,脱溶剂气温度450 ℃,脱溶剂气体积流量900 L/h,锥孔气体积流量50 L/h,碰撞能量(CE)10~45 eV, LockMass:亮氨酸脑啡肽(*m/z* 554.2615)。

1.2.4 数据处理

分别对经UPLC-Q-TOF-MS正离子和负离子模式采集的数据进行预处理。将. raw原始数据导入Progenesis QI软件后,进行峰识别、峰对齐、基线校正、去卷积和归一化等预处理,获得三维数据矩阵。将数据导入EZInform软件中,对样本间不符合正态分布的数据进行log转换,以Hotelling’s T2 range algorithmin最小95%置信区间对数据进行验证,删除异常点,采用帕莱托标度换算^[21]^,进行主成分分析(principle clustering analysis, PCA)和偏最小二乘法判别分析(partial least square discriminant analysis, PLS-DA)。根据精确前体离子和碎片离子质量信息检索Chemspider、HMDB数据库推测化合物结构。

## 2 结果与讨论

### 2.1 数据质量控制与SPE前处理方法的适用性评价

为了获得真实可靠的数据,本研究从以下两方面进行数据质量控制:一是分别在正、负离子采集分析时,采用组间穿插进样、组内随机进样的方式进行检测;二是通过绘制QC样品的PCA得分图考察检测系统的稳定性,如图1所示,正、负离子模式下蜂蜜QC样品多次进样的峰面积偏差在-2SD~2SD之间。结果表明,色谱系统的分离性能和稳定性良好,分析方法稳定、可靠。

**图1:**
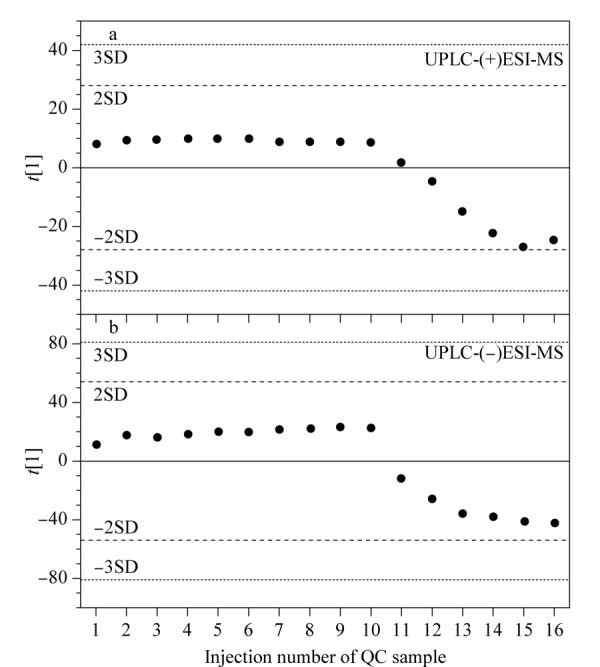
基于蜂蜜质控样品的UPLC-(+)ESI-MS和UPLC-(-)ESI-MS数据构建的主成分分析(PCA)模型得分图

文献^[3,4,5,6]^中报道蜂蜜中的微量成分如黄酮、酚酸和小分子物质可作为溯源标志物,因此本研究基于前期工作^[10,22]^所建立的固相萃取方法去除蜂蜜中大量的糖从而富集微量成分,以加标回收率为评价指标,对蜂蜜中黄酮、酚酸与脱落酸等38个化合物进行了定量分析,加标回收率为69.1%~108%,说明该固相萃取方法适用于对蜂蜜中的主要微量成分进行富集和前处理。

### 2.2 UPLC-Q-TOF-MS分析结果

正、负离子模式下不同蜜源植物单花蜜的UPLC-Q-TOF-MS总离子色谱图如图2所示,从轮廓图中可见整体轮廓图存在不同样品间的差异,但部分样品间色谱图具有一定相似度,为了揭示不同来源单花蜜间组分的差异,进一步采用多维模式识别,对反映样本的多个变量(峰)进行观测,从各个角度收集数据信息以便进行较为全面的分析。

**图2:**
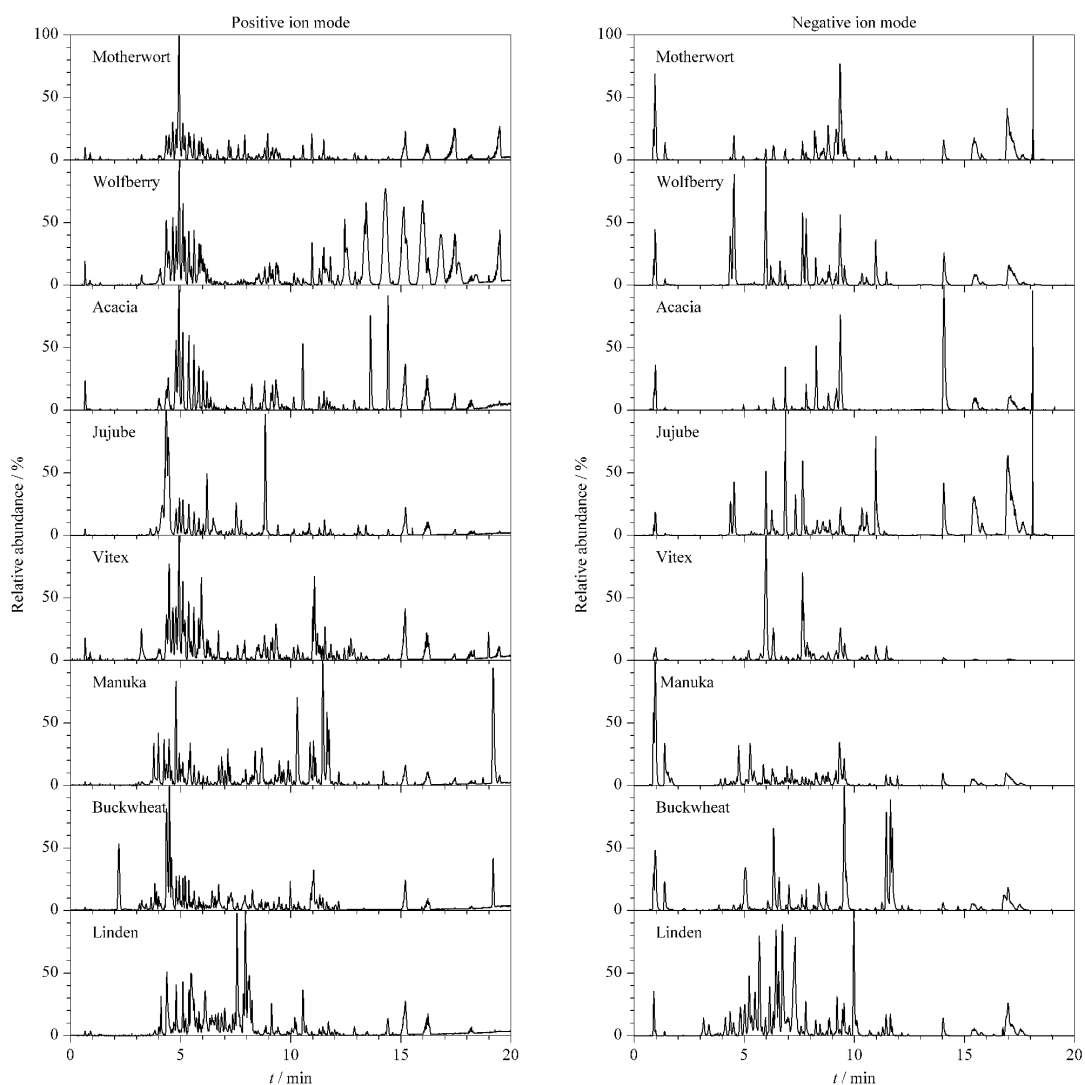
8种蜂蜜UPLC-Q-TOF-MS^E^正离子和负离子检测模式下的总离子流色谱图

### 2.3 不同来源单花蜜的代谢判别分析

2.3.1 主成分分析

采用PCA对8种蜜源植物的单花蜜进行差异分析,对UPLC-Q-TOF-MS正离子和负离子模式采集数据分别进行分析,数据由Progenesis QI分析处理后分别得到10557和2706个变量,将其质荷比、保留时间和峰面积组成的三维矩阵分别导入EZinform软件进行PCA分析。

正离子模式下8种蜂蜜的PCA分析显示前3个主成分共解释了48.05%的原始变量信息(PC1: 30.5%, PC2: 11.5%, PC3: 6.05%), PCA得分图如图3a所示:在第一主成分和第二主成分的分值图上,椴树蜜和枣花蜜分别分布在第一象限和第三象限,呈明显分离;荞麦蜜和麦卢卡蜜分布在第四象限,和其他蜜具有显著差异;洋槐蜜、荆条蜜、益母草蜜和枸杞蜜分布在第二象限,其中洋槐蜜和荆条蜜分别聚类,益母草蜜和枸杞蜜之间无明显差异。

**图3:**
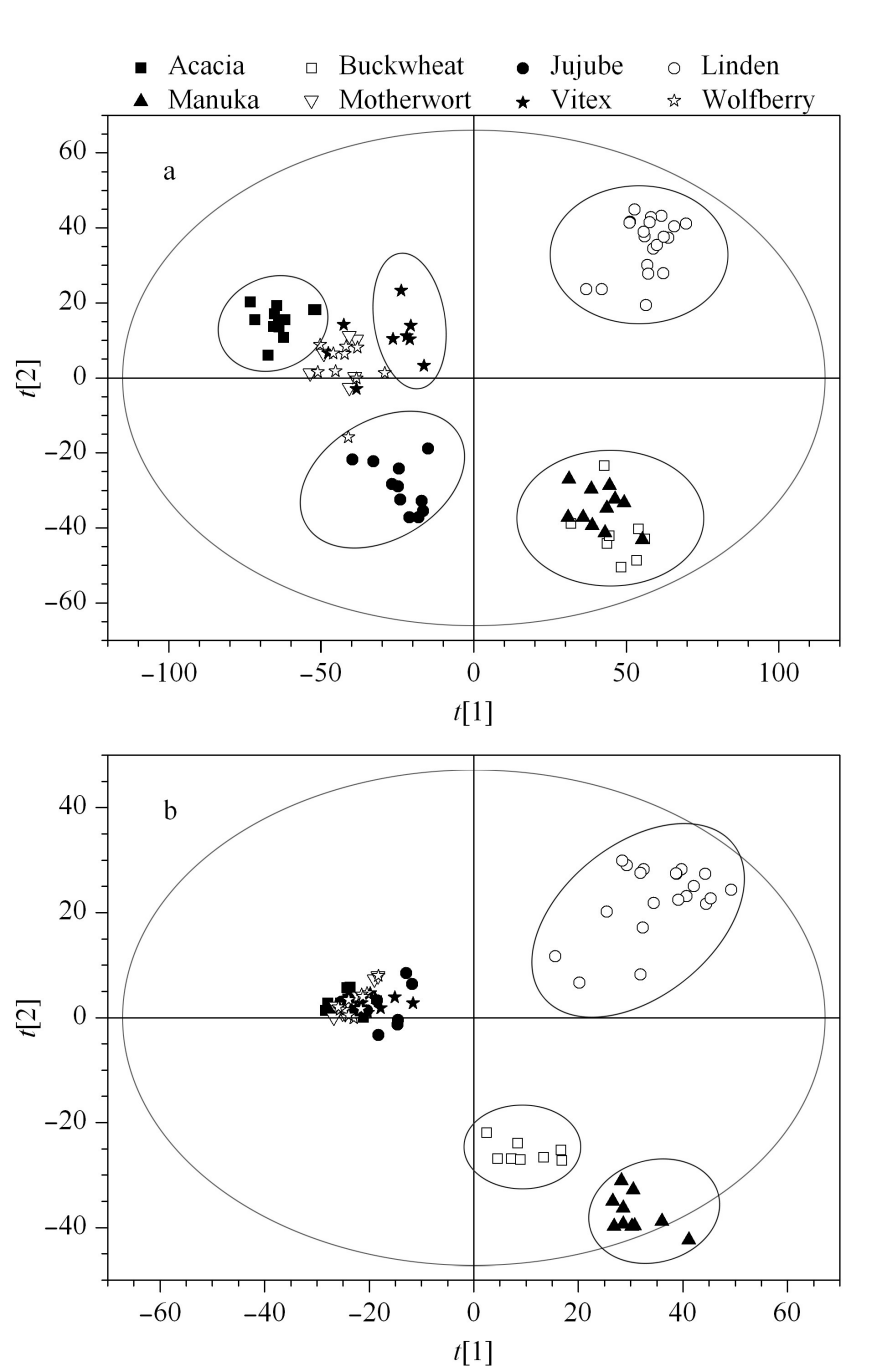
基于8种蜂蜜的(a)UPLC-(+)ESI-MS和(b)UPLC-(-)ESI-MS数据构建的PCA模型得分图

负离子模式下8种蜂蜜样本的PCA分析显示前3个主成分共解释了57.88%的原始变量信息(PC1: 32.8%, PC2: 17.3%, PC3: 7.78%), PCA得分图如图3b所示:在第一主成分和第二主成分的分值图上,椴树蜜、荞麦蜜和麦卢卡蜜样本呈现明显分离,其中荞麦蜜和麦卢卡蜜分别聚类有差异。

以上主成分分析说明椴树蜜、荆条蜜、荞麦蜜、麦卢卡蜜、枣花蜜和洋槐蜜在代谢成分上存在明显的差异;而枸杞蜂蜜和益母草蜂蜜相似度较高,未能明显区分;显微镜下观察枸杞蜂蜜和益母草蜂蜜中药材花粉含量约30%,彼此次生代谢产物的差异性较弱,但仍可以与其他单花蜜区分。

2.3.2 PLS-DA分析及模型验证

进一步采用PLS-DA分析对构建的模型进行验证,对不同来源单花蜜的差异进行判别分析。分别通过内部验证和外部验证两种方法对模型进行识别和验证。内部验证通过模型拟合的*Q*^2^值表示模型的预测能力,如图4所示,正离子模式下所建模型对8种蜂蜜的判别解释能力达96.2% (*R*^2^*Y*=0.962),对未知样本的预测能力为89.1%(*Q*^2^=0.891);负离子模式下所建模型对8种蜂蜜的判别解释能力达94.9% (*R*^2^*Y*=0.949),对未知样本的预测能力为81.3%(*Q*^2^=0.813)。

**图4:**
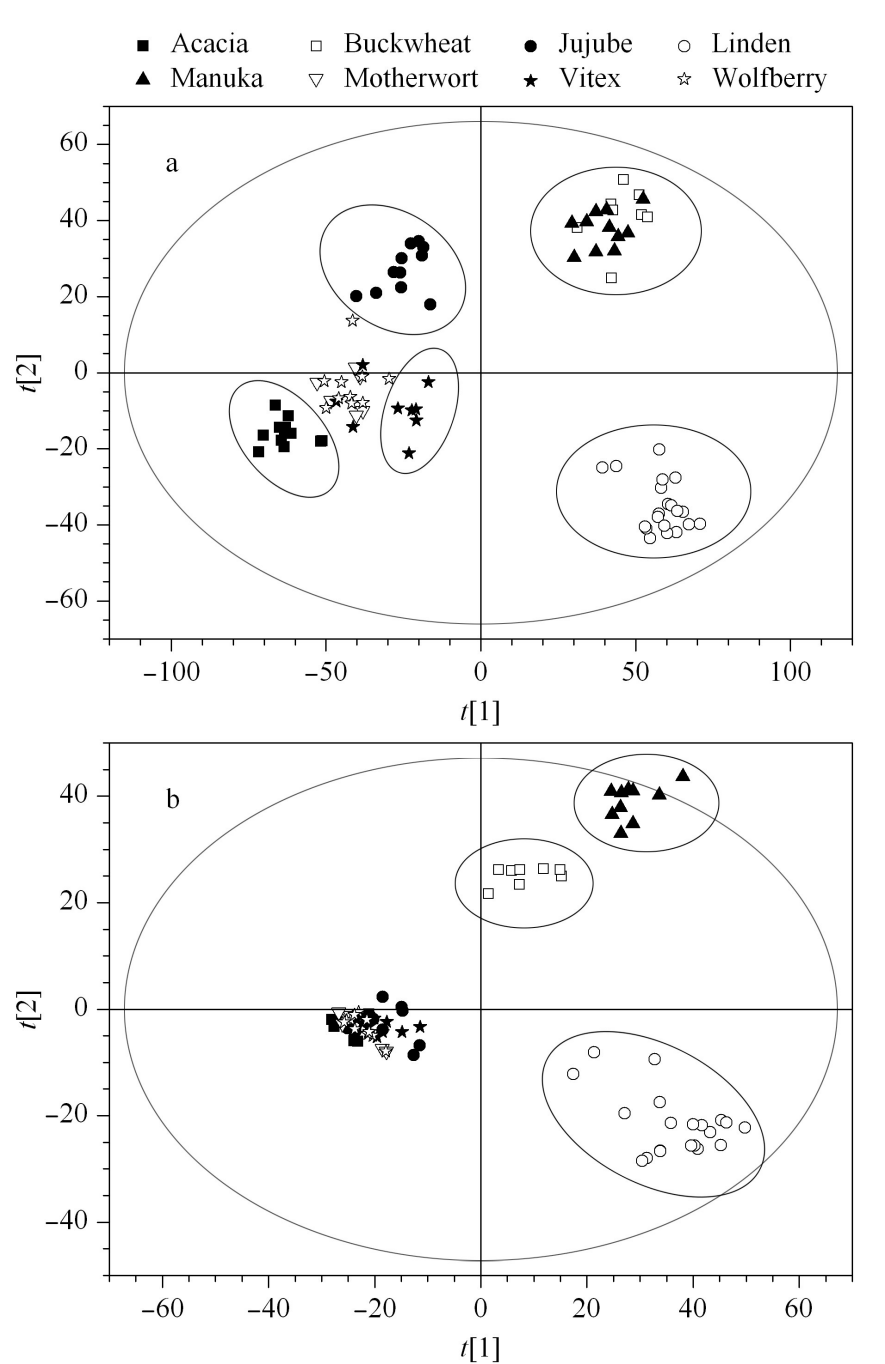
基于8种蜂蜜的(a)UPLC-(+)ESI-MS和(b)UPLC-(-)ESI-MS数据构建的偏最小二乘法判别分析(PLS-DA)模型得分图

采用置换测试法(permutation test)对模型进行外部交叉验证(*n*=200),正、负离子模式交互排列模型验证结果如图5a(*R*^2^=0.360, *Q*^2^=-0.684)和图5b(*R*^2^=0.231, *Q*^2^=-0.529)所示,回归线斜率大,与纵轴的截距小,表明模型未过拟合且稳健,具有很好的稳定性和预测性^[23]^。

**图5:**
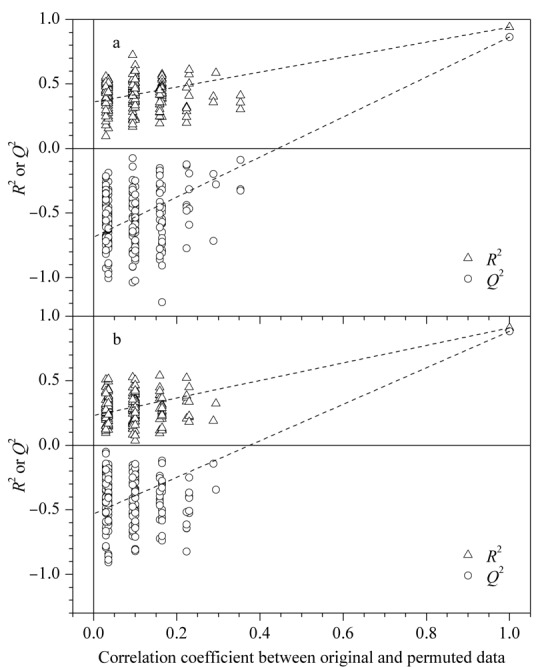
基于(a)UPLC-(+)ESI-MS和(b)UPLC-(-)ESI-MS数据构建的置换测试验证图

### 2.4 差异代谢物的筛选及鉴别

差异代谢产物的筛选结合3个标准:变量重要性投影(variable importance in project, VIP)>1, Anova方差分析*p*<0.05,最大差异倍数值(max folder change)>1.5,根据PLS-DA模型的变量重要性投影与系数图筛选,以横坐标为相关系数,纵坐标为变量重要性投影,每个点代表一个变量,如图6所示,再参照每个变量在不同样本的丰度图来筛选潜在差异代谢物。差异代谢产物经过筛选后基于前体离子的精确质量数、前体离子同位素组成以及碎片离子的信息进行元素组成的确定,设定分子式的质量误差小于5×10^-6^,基于前体离子质谱信息、质谱碎片的特征裂解方式以及碎片峰匹配情况检索在线数据库(Chemspider、HMDB等)并对鉴定的化合物进行前体离子与碎片离子的匹配评分(score),鉴定结果如表1所示,共鉴定出32个蜂蜜代谢差异化合物,其中黄酮类化合物17个、酚酸类化合物6个、苯苷与萜苷类化合物6个、其他类化合物3个。

**图6:**
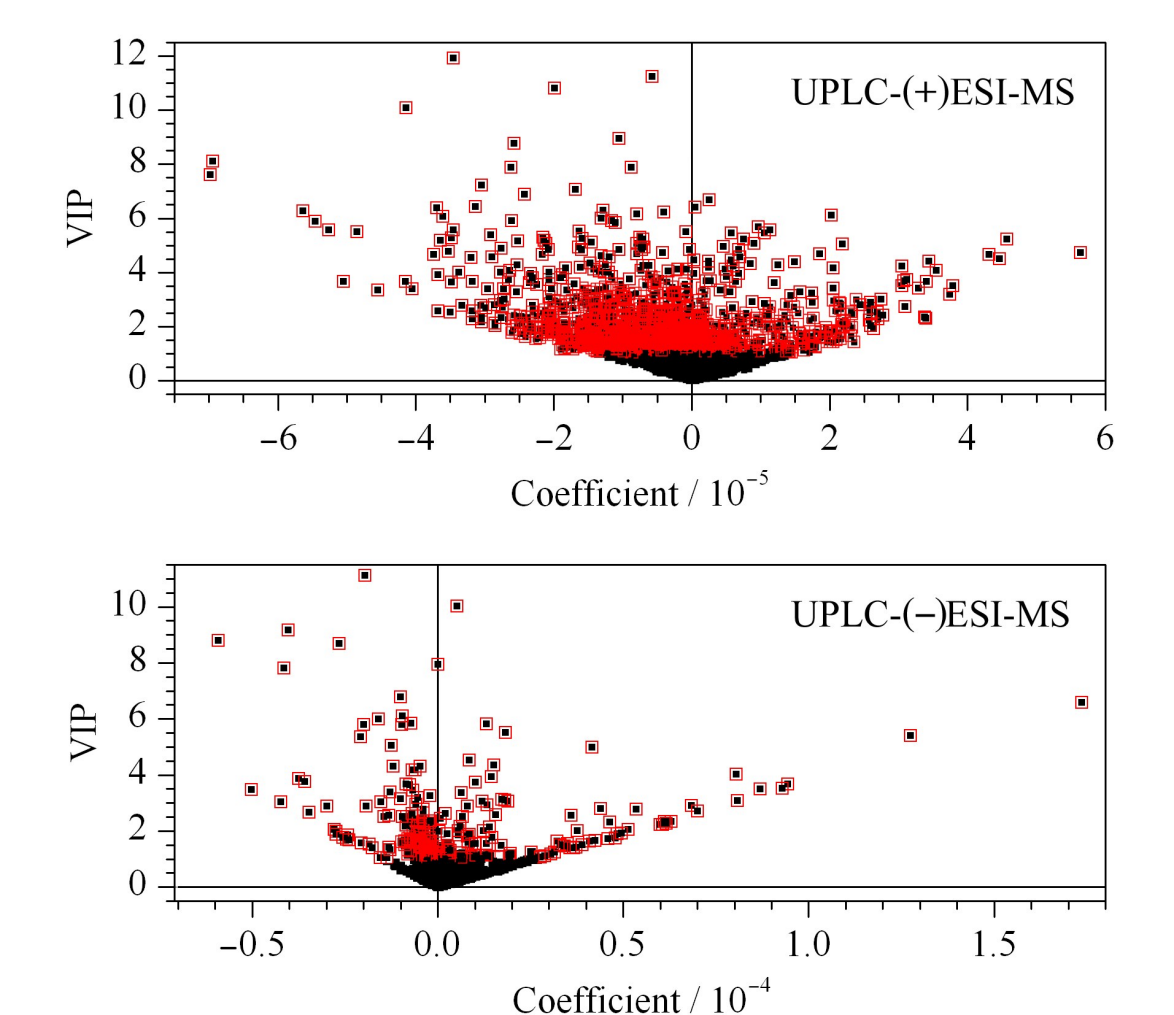
基于8种蜂蜜UPLC-(+)ESI-MS和UPLC-(-)ESI-MS数据构建的PLS-DA模型图

**表1:** 基于UPLC-Q-TOF-MS在ESI(-)/ESI(+)模式下初步鉴定的潜在蜂蜜标志物

No.	RT^a^/ min	Precursor ion								Fragment ion (*m/z*)			Tentative identification				Score (*F* score^b^)			Highest mean				Lowest mean		
		*m/z*		Ion		Elemental composition		Mass error/mDa																		
1	3.20	265.0355		M-H		C_12_H_10_O_7_		0.7		206.0821		2-*O*-*p*-coumaroyltartronic acid				37.7				buckwheat			vitex			
2	4.66	549.1827		M+FA-H		C_22_H_32_O_13_		0.8		503.1770						46.8				linden			manuka			
										487.1457						-51.8										
										323.0984																
3	4.80	393.1762		M-H_2_O-H		C_17_H_32_O_11_		0.2		365.1812		isopentyl gentiobioside				40.0				linden			buckwheat			
										347.1708						-24.5										
										205.0713																
										137.0243																
4	5.15	593.1525		M-H		C_27_H_32_O_16_		1.9		473.1109		apigenin-7-[galactosyl-(1→4)-				37.9				vitex			manuka			
										341.1088		mannoside]				-10.2										
										121.0290																
5	5.27	445.1706		M-H		C_15_H_20_O_8_		0.3		327.1086		crosatoside B				48.9				linden			wolfberry			
										295.1198						-60.0										
6	5.29	507.2082		M-H		C_22_H_36_O_13_		0.4		445.1700		6-*O*-oleuropeoylsucrose				44.1				linden			manuka			
										323.0988						-32.5										
										221.0670																
										183.1027																
7	5.65	739.2100		M-H		C_33_H_40_O_19_		1.5		593.1532		kaempferol 3-(2″-rhamnosylru-				42.1				acacia			buckwheat			
										447.0950		tinoside)				-40.1										
8	6.18	163.0401		M-H		C_9_H_8_O_3_		0.6		119.0502		phenylpyruvic acid				56.3				buckwheat			acacia			
																-88.2										
9	6.00	367.1036		M-H		C_17_H_20_O_9_		0.7		263.0946		3-*O*-caffeoyl-1-*O*-methylquinic				42.3				vitex			manuka			
										179.0363		acid				-21.0										
										135.0452																
10	6.34	367.1037		M-H		C_17_H_20_O_9_		0.8		147.0451		3-*O*-caffeoyl-4-*O*-methylquinic				38.4				vitex			manuka			
										135.0452		acid/3-feruloylquinic acid				-2.9										
11	6.54	401.1454		M-H		C_18_H_26_O_10_		0.6		369.1202		benzyl *O*-[arabinofuranosyl-				39.6				linden			buckwheat			
										292.0948		(1→6)-glucoside]				-10.2										
12	7.44	557.1317		M-H_2_O-H		C_27_H_28_O_14_		2.2		431.0991		vitexin 6″-(3-hydroxy-3-methyl-				46.8				vitex			manuka			
										395.0987		glutarate)				-56.6										
										135.0450																
13	7.61	297.0779		M-H		C_17_H_14_O_5_		1.6		203.0364		2-(3-hydroxy-4,5-dimethoxyphe-				41.3				buckwheat			vitex			
										151.0401		nyl)-4*H*-chromen-4-one				-22.2										
14	7.62	237.0923		M-H_2_O-H		C_16_H_16_O_3_		0.8		151.0771		7-hydroxy-5-methoxyflavan				39.8				buckwheat			vitex			
										143.0504						-10.8										
										131.0508																
15	8.10	529.1360		M-H		C_26_H_26_O_12_		1.4		367.1044		4-*O*-caffeoyl-3-*O*-feruloylquinic acid/				37.4				vitex			acacia			
										161.0262		1-feruloyl-5-caffeoylquinic acid				-7.7										
										135.0451																
16	8.23	431.0996		M-H		C_21_H_20_O_10_		0.8		343.0842		6-*C*-fucosylluteolin				39.5				acacia			buckwheat			
										284.0338						-17.5										
17	8.46	301.0355		M-H		C_15_H_10_O_7_		0.7		151.0037		quercetin				42.6				manuka			acacia			
																(25.2)^c^										
18	8.58	567.2445		2M-H		C_14_H_20_O_6_		0.4		119.0502		2-phenylethyl-*β*-*D*-glucopyranoside				39.1				linden			buckwheat			
																-0.1										
19	8.60	237.0919		M-H_2_O-H		C_16_H_16_O_3_		0.4		163.0765		xenognosin A				41.0				buckwheat			vitex			
										119.0503						-16.6										
20	8.86	281.0825		M-H_2_O-H		C_17_H_16_O_5_		1.1		235.0765		3'-methoxydihydroformononetin				40.3				buckwheat			vitex			
										207.0662						-14.9										
										163.0764																
No.	RT^a^/ min		Precursor ion								Fragment ion (*m/z*)				Tentative identification				Score (*F* score^b^)			Highest mean				Lowest mean
			*m/z*		Ion		Elemental composition		Mass error/mDa																	
21	9.24		281.0822		M-H_2_O-H		C_17_H_16_O_5_		0.8		237.0923			2-hydroxy-3,4-diphenylpentanedioic				47.8			buckwheat				vitex	
											235.0760			acid				-51.9								
22	10.00		339.0879		M+FA-H		C_18_H_14_O_4_		0.6		307.0618			3-methoxy-2-(4-methylbenzoyl)-				46.2			buckwheat				vitex	
											263.0717			4*H*-chromen-4-one				-42.3								
23	10.28		283.0619		M-H		C_16_H_12_O_5_		1.3		239.0361			galangin 3-methyl ether				44.6			manuka				jujube	
											211.0419							-37.5								
24	10.56		315.0515		M-H		C_16_H_12_O_7_		1.0		165.0201			pollenitin				39.3			manuka				vitex	
											92.0263							-11.2								
25	10.72		279.0667		M-H_2_O-H		C_17_H_14_O_5_		1.0		251.0723			7-hydroxy-6-methoxy-3-(4-me-				41.7			buckwheat				linden	
											250.0654			thoxyphenyl)-4*H*-chromen-4-one				-23.6								
26	11.55		283.0619		M-H		C_16_H_12_O_5_		1.3		268.0387			5,7-dihydroxy-2-(3-methoxyphe-				54.7			buckwheat				jujube	
														nyl)-4*H*-chromen-4-one				-87.2								
27	11.59		285.0770		M-H		C_16_H_14_O_5_		0.8		243.0669			sakuranetin				39.6			buckwheat				acacia	
											136.0166							(10.4)^d^								
28	11.81		299.0565		M-H		C_16_H_12_O_6_		1.0		284.0330			luteolin 7-methyl ether				41.0			buckwheat				acacia	
											161.0250							-18.2								
29	11.88		313.0720		M-H		C_17_H_14_O_6_		0.7		253.0511			gnaphaliin				48.1			manuka				vitex	
											271.0600							-51.3								
											281.0836															
30	12.22		313.0721		M-H		C_17_H_14_O_6_		0.6		298.0479			7-hydroxy-2-(4-hydroxy-3,5-dime-				44.9			buckwheat				jujube	
											253.0510			thoxyphenyl)-4*H*-chromen-4-one				-36.9								
											163.0407															
31	5.89		812.2626		M+H+ACN		C_34_H_41_O_19_		-1.3		795.2551			isorhamnetin 3-rutinoside 4'-rham-				26.6			jujube				acacia	
											705.2469			noside				-11.2								
											658.2361															
											289.0338															
32	6.24		591.3534		M+H-2H_2_O		C_33_H_54_O_11_		-0.1		284.1420			ponasteroside A				37.7			jujube				linden	
																		-1.94								

a. retention time; b. fragmentation score; c. confirmed by the standard of quercetin; d. confirmed by the standard of sakuranetin.

研究发现荞麦蜜和麦卢卡蜜中含有种类丰富的黄酮类化合物,其中槲皮素、樱花亭、木犀草素-7-甲基醚、茶花粉黄酮、4',7-二羟基-3',5'-二甲氧基-黄酮(7-hydroxy-2-(4-hydroxy-3,5-dimethoxyphenyl)-4*H*-chromen-4-one)和5,7-二羟基-3'-甲氧基-黄酮(5,7-dihydroxy-2-(3-methoxyphenyl)-4*H*-chromen-4-one)等化合物含量高,与其他种类蜂蜜具有显著性差异,且槲皮素和樱花亭经对照品进一步确证,课题组曾在相关研究中进行过定量测定及含量对比^[22]^;此外3-甲氧基-2-(4-甲基苯甲酰基)-4*H*-1-苯并吡喃-4-酮(3-methoxy-2-(4-methylbenzoyl)-4*H*-chromen-4-one)、2-羟基-3,4-二苯基戊二酸、3'-甲氧基二氢芒柄花素、苯丙酮酸、酒石酸-2-*O*-对香豆酰酯、2'-羟基-3',4'-二甲氧基-黄酮(2-(3-hydroxy-4,5-dimethoxyphenyl)-4*H*-chromen-4-one)、6,4'-二甲氧基-7-羟基-异黄酮(7-hydroxy-6-methoxy-3-(4-methoxyphenyl)-4*H*-chromen-4-one)、xenognosin A和7-羟基-5-甲氧基黄烷在荞麦蜜中含量高,可作为荞麦蜜的特征代谢产物;鼠曲草黄素和高良姜素3-*O*-甲醚在麦卢卡蜜中含量高。进一步研究发现椴树蜜的特征性化合物为苯苷类(*S*)-2-甲基-1-[2-芹菜糖基-(1→6)-葡萄糖基-4,6-二羟基苯基]丁烷-1-酮((*S*)-multifidol 2- [apiosyl- (1→6)-glucoside])、2-苯乙基-*β*-*D*-吡喃葡萄糖苷、苯甲醇*O*-[呋喃阿拉伯糖基-(1→6)-呋喃葡萄糖苷]、西红花新苷乙和萜苷类异戊基龙胆双糖苷、6-[4-(1-羟基-1-甲基乙基)-1-环己烯-1-羧酸盐]蔗糖(6-*O*-oleuropeoylsucrose);荆条蜜特征性地富含奎宁酸衍生物4-*O*-咖啡酰-3-*O*-阿魏酰奎宁酸或1-阿魏酰-5-咖啡酰奎宁酸、3-*O*-咖啡酰-4-*O*-甲基奎宁酸或3-阿魏酰奎宁酸、3-*O*-咖啡酰-1-*O*-甲基奎宁酸以及黄酮类化合物牡荆素6'-(3-羟基-3-甲基戊二酸)和芹菜素-7-*O*-[半乳糖基-(1→4)-甘露糖苷], 木犀草素-6-*C*-岩藻糖苷和山柰酚-3-*O*-2″-鼠李糖基芸香糖苷在洋槐蜜中含量高,Truchado等^[24]^曾报道过蜂蜜中含有黄酮己糖和戊糖碳苷类化合物,文献^[6]^曾报道刺槐蜂蜜中含有的一系列山柰酚的鼠李糖己糖苷类化合物。

## 3 结论

本研究建立了基于UPLC-Q-TOF-MS对我国市场上多种不同来源的主流单花蜜(洋槐蜂蜜、枣花蜂蜜、荆条蜂蜜、椴树蜂蜜、荞麦蜂蜜、麦卢卡蜂蜜、枸杞蜂蜜、益母草蜂蜜)中差异次生代谢产物的非靶向代谢组学分析方法,方法快速有效,具有分析时间短、专属性好、稳定性高等优点。该模型可以理想地将枣花蜂蜜、椴树蜂蜜、荞麦蜂蜜、麦卢卡蜂蜜、洋槐蜂蜜和荆条蜂蜜彼此区分。方法不仅体现了蜂蜜的化学多样性,而且更加丰富了蜂蜜溯源识别的标志化合物群,其中黄酮与酚酸类化合物不仅是蜂蜜中的活性组分群^[22]^,而且可作为蜂蜜蜜源判别的差异标志物。本研究建立的蜂蜜溯源分析方法可以扩展到多种不同蜜源单花蜜的区分识别,为不同单花蜜的质量评价建立有效方法,为单花蜜的品质分析与规范蜂产品市场提供技术支持。

## References

[1] Chinese Pharmacopoeia Commission. Pharmacopoeia of the People’s Republic of China. Part 1. Beijing: China Medical Science Press, 2020: 374

[2] Zhang F X. Modern Beekeeping. Beijing: China Agricultural University Press, 1998: 285

[3] Fechner D C, Hidalgo M J, Ruiz Díaz J D, et al. Food Biosci, 2020,33:100483

[4] Shen S, Zhang S Q. Chinese Journal of Antibiotics, 2016,41(11):810

[5] Tomás-Barberán F A, Martos I, Ferreres F, et al. J Sci Food Agric, 2001,81:485

[6] Truchado P, Ferreres F, Bortolotti L. J Agric Food Chem, 2008,56:8815 18729455 10.1021/jf801625t

[7] Wang Q, Zhao H A, Xue X F, et al. Food Chem, 2020,309:12656 10.1016/j.foodchem.2019.125656 31699558

[8] Zhao J, Du X J, Cheng N, et al. Food Chem, 2016,194:167 26471540 10.1016/j.foodchem.2015.08.010

[9] Wang Q, Xue X J, Zhao J. Journal of Agricultural Science and Technology, 2013,15(4):42

[10] Shen S, Wang J B, Zhuo Q, et al. Molecules, 2018,23(5):1110 10.3390/molecules23051110 PMC6099688 29738446

[11] Jandríc Z, Frew R D, Fernandez-Cedi L N, et al. Food Control, 2017,72:189

[12] Shen C Y, Guo S Y, Ding T, et al. Chinese Journal of Chromatography, 2017,35(10):1068 29048804 10.3724/SP.J.1123.2017.07009

[13] Yan L J, Xu D M, Xue X F, et al. Chinese Journal of Chromatography, 2019,37(6):589 31152508 10.3724/SP.J.1123.2018.12011

[14] Nasab S G, Yazd M J, Marini F, et al. Chemometr Intell Lab, 2020,202:104037

[15] Aykas D P, Shotts M L, Rodriguez-Saona L E. Food Control, 2020,117:107346

[16] He C X, Liu Y, Liu H I, et al. Food Res Int, 2020,130:108936 32156383 10.1016/j.foodres.2019.108936

[17] Song X Y, She S, Xin M M, et al. Food Compos Anal, 2020,86:103390

[18] Chen H, Fan C L, Chang Q Y, et al. J Agric Food Chem, 2014,62(11):2443 24579819 10.1021/jf405045q

[19] Soares S, Amaral J S, Oliveira M B P P, et al. Food Control, 2015,48:130

[20] Yu Q H, Zhang J K, Ye X Q, et al. Chinese Journal of Chromatography, 2016,34(7):657

[21] Qi X Q, Wang Y L, Chen X Y. Plant Metabolomics: Methods and Applications. 1st ed. Beijing: Chemical Industry Press, 2011: 102

[22] Shen S, Wang J B, Chen X, et al. Food Chem, 2019,293:169 31151598 10.1016/j.foodchem.2019.04.105

[23] Slupsky C M, Rankin K N, Wagner J, et al. Anal Chem, 2007,79(18):6995 17702530 10.1021/ac0708588

[24] Truchado P, Vit P, Ferreres F, et al. J Chromatogr A, 2011,1218:7601 21831383 10.1016/j.chroma.2011.07.049

